# Considerations to Be Taken When Carrying Out Medicinal Plant Research—What We Learn from an Insight into the IC_50_ Values, Bioavailability and Clinical Efficacy of Exemplary Anti-Inflammatory Herbal Components

**DOI:** 10.3390/ph14050437

**Published:** 2021-05-06

**Authors:** Mona Abdel-Tawab

**Affiliations:** 1 Central Laboratory of German Pharmacists, Carl-Mannich-Str. 20, 65760 Eschborn, Germany; tawab@em.uni-frankfurt.de; Tel.: +49-6196-937-955; 2 Institute of Pharmaceutical Chemistry, Johann Wolfgang Goethe University, Max-von-Laue-Straße 9, 60438 Frankfurt am Main, Germany

**Keywords:** medicinal plant, boswellic acids, curcumin, quercetin, resveratrol, bioavailability, tissue-plasma-ratio, tissue distribution, metabolomics, combinatorial approaches

## Abstract

Medicinal plants represent a big reservoir for discovering new drugs against all kinds of diseases including inflammation. In spite the large number of promising anti-inflammatory plant extracts and isolated components, research on medicinal plants proves to be very difficult. Based on that background this review aims to provide a summarized insight into the hitherto known pharmacologically active concentrations, bioavailability, and clinical efficacy of boswellic acids, curcumin, quercetin and resveratrol. These examples have in common that the achieved plasma concentrations were found to be often far below the determined IC_50_ values in vitro. On the other hand demonstrated therapeutic effects suggest a necessity of rethinking our pharmacokinetic understanding. In this light this review discusses the value of plasma levels as pharmacokinetic surrogates in comparison to the more informative value of tissue concentrations. Furthermore the need for new methodological approaches is addressed like the application of combinatorial approaches for identifying and pharmacokinetic investigations of active multi-components. Also the physiological relevance of exemplary in vitro assays and absorption studies in cell-line based models is discussed. All these topics should be ideally considered to avoid inaccurate predictions for the efficacy of herbal components in vivo and to unlock the “black box” of herbal mixtures.

## 1. Introduction

Chronic inflammation is associated with many chronic diseases like arthritis, asthma, atherosclerosis, autoimmune diseases, diabetes, allergy, cancer, and conditions of aging [1]. Moreover it has been recognized as the greatest threat to human health, with more than 50% of all deaths being attributable to inflammation-related diseases such as ischemic heart disease, stroke, cancer, diabetes mellitus, chronic kidney disease, non-alcoholic fatty liver disease (NAFLD) and autoimmune and neurodegenerative conditions [2]. In spite of extensive research activities devoted to the discovery of new anti-inflammatory drugs evidence based anti-inflammatory therapies are still restricted to the administration of non-steroidal anti-inflammatory drugs (NSAIDs), corticosteroids and recently cytokine blocking biologicals. Unfortunately these existing anti-inflammatory treatment options are associated with a lot of side effects [3]. Therefore a large portion of research activities in academia and industry is still devoted to the discovery of new anti-inflammatory compounds that are urgently needed [4].

Inflammation is a result of activated cellular elements and the existence of various biochemical mediators like cytokines (e.g., tumor necrosis factor-α (TNFα), interleukin-(IL)-1β, IL-2, IL-6, IL-8), kinases (e.g., p38 kinase, c-Jun-N-terminal kinases (JNKs), extracellular-signal regulated kinases (ERKs), mitogen-activated protein (MAP) kinase), transcription factors (e.g., nuclear factor-kappa beta (NF-κB) and matrix metalloproteinases (MMPs). Also the pro-inflammatory activity of lipid derived mediators e.g., the leukotrienes formed by lipoxygenases (LO) and prostaglandins by cyclooxygenases (COX) are well known. NF-κB is considered to be the chief regulator of the inflammatory response as it regulates the transcription of inflammatory cytokines like IL-1β, IL-2, IL-6, IL-8 and TNFα along with the genes encoding for COX-2, inducible nitric oxide synthase (iNOS, a key enzyme in the macrophage inflammatory response) and cell adhesion molecules. Typically NF-κB is localized to the cytoplasm by the inhibitory protein IκB. Upon stimulation IκB protein is phosphorylated by IκB kinase (IKK), ubiquitinated, and degraded. This allows the free NF-κB to accumulate in the nucleus, where it can activate transcription [3]. Through all ages and in all cultures medicinal plants represented a big reservoir for a large number of drugs against all kinds of diseases including inflammation [4]. In fact various medicinal plant extracts and their identified/isolated active phytoconstituents have been reported to exhibit potential anti-inflammatory activity by interaction with inflammatory pathways or specifically with certain components of the pathways like e.g., the proinflammatory mediator production [3]. However research on medicinal plants has proved to be very difficult. This is attributed to the complex composition of medicinal herbs, being composed of numerous chemical constituents each being identical to a single component chemical drug. Although much effort has been invested in identifying as many constituents as possible, a full understanding of all chemical structures and their contents in a single herb is still not available [5]. The complex interplay of the individual constituents resulting in promising therapeutic effects also remains unresolved to a great extent. Nevertheless preclinical research, that is in vitro, cell-based and animal studies on the pharmacological action of a large number of promising anti-inflammatory medicinal plants and their individual constituents, are available in an unconceivable number [4], but as far as we know, most of the herbal constituents are poorly absorbed or extensively metabolized resulting in very low bioavailability following oral administration [5]. In a word, appropriate plasma concentrations in a similar high range like those applied in preclinical research could hardly be achieved for most medicinal plants [5]. At the same time sound clinical trials are often missing [4] and hence a profound judgement on the efficacy of promising anti-inflammatory medicinal plants is not possible in most cases.

Apart from this unfortunate situation, medicinal plants like *Boswellia serrata* and plant-derived compounds like curcumin, quercetin, and resveratrol, are the subject of hype in the media, even in scientific circles, for their anti-inflammatory potential [4]. In fact oleogum resin of *Boswellia serrata* represents one of the best investigated medicinal plants beyond those plants already authorised as herbal medicinal products, e.g., Ginkgo or St. John’s Wort. The purified and isolated phytoconstituents curcumin, and the widely spread quercetin and resveratrol have also been extensively studied. Therefore they were chosen as well studied examples for discrepancies often observed between the pharmacologically active concentrations determined in vitro and the concentrations actually determined in human plasma. For that purpose the hitherto known pharamcologically active concentrations expressed as IC_50_ values determined in in vitro assays, bioavailability, and clinical efficacy with regard to diseases associated with inflammation are revised. Based on the insights gained the need for an inevitable paradigm change in interpreting pharmacokinetic results as well as conducting medicinal plant research is adressed. Furthermore powerful tools that can help better judging the potential of medicinal plants in a very early stage of research more easily and more effectively will be discussed. In order not to go beyond this scope in vitro and in vivo studies refering to the mechanisms of anti-inflammatory action will not be in the focus of this review.

## 2. Frankincense Extracts: In Vitro IC_50_ Values, Bioavailability and Clinical Efficacy

*Boswellia serrata* counts among the most intensively studied anti-inflammatory medicinal plants with more than 650 publications recorded in the PubMed literature database up to now. In folk medicine lipophilic frankincense extracts are used as alternatives to anti-inflammatory steroidal drugs (i.e., glucocorticoids) or nonsteroidal anti-inflammatory drugs (NSAIDs) for the treatment of diseases associated with inflammation like rheumatoid arthritis, osteoarthritis, asthma, atopic dermatitis, and inflammatory bowel diseases. A detailed overview of a plethora of articles addressing the modes of action of the anti-inflammatory effect both in vitro and in vivo is given in the review of Efferth and Oesch [6].

**Figure 1:**
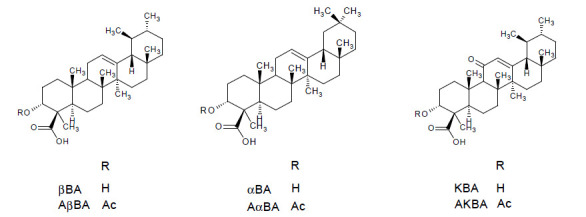
Chemical structures of the most relevant boswellic acids: β-boswellic acid (βBA), α-boswellic acid (αBA), 11-keto-β-boswellic acid (KBA), 3-O-acetyl-β-boswellic acid (AβBA), 3-O-acetyl-α-boswellic acid (AαBA), 3-O-acetyl-11-keto-β-boswellic acid (AKBA).

In summary *Boswellia serrata* represents one of five species from the genus *Boswellia* (family: Burseraceae) commonly known as frankincense. This term refers to the oleogum resin of *B. serrata* Roxb., *B. carterii* Birdw., *B. sacra* Flueck, *B. papyrifera* Hochst, and *B. frereana* Birdw. The main components of the gum resin of *Boswellia serrata* are 10% volatile oils, 60% lipophilic resin, and 30% hydrophilic gums [7]. The anti-inflammatory effects are mainly attributed to the boswellic acids, i.e., 11-keto-β-boswellic acid (KBA), 3-O-acetyl-11-keto-β-boswellic acid (AKBA), α-boswellic acid (αBA), β-boswellic acid (βBA), 3-O-acetyl-α-boswellic acid (AαBA), 3-O-acetyl-β-boswellic acid (AβBA) [8]. The chemical structures are presented in Figure 1. Although more than 12 different types of boswellic acids have been identified in the gum resin of *B. serrata* and *B. carterii*, mostly the above mentioned six boswellic acids received considerable pharmacological attention [9]. This may be attributed to their high occurrence in lipophilic frankincense extracts, reaching 14 to 25% (m/m) in case of KBA, AKBA, βBA and AβBA [10]. Some additional chemical components include lupeolic acid, roburic acids, tirucallic acids as well as incensole acetate, incensole oxide and isoincensole oxides, just to name a few [9].

### 2.1. In Vitro IC_50_ Values

It is believed that the anti-inflammatory effects of boswellic acids are caused by different mechanisms of action including inhibition of key enzymes, kinases and transcription factors involved in the initiation and maintenance of inflammation [9]. For example boswellic acids were reported to inhibit the key enzymes 5-lipoxygenase (5-LO), cyclooxygenase-2 (Cox-2), and microsomal prostaglandin E_2_ synthase-1 (mPGES-1). The pro-inflammatory prostaglandins (PG) and leukotrienes (LT) produced by the Cox-2 and 5-LO pathways from arachidonic acid significantly contribute to the inflammatory response [10]. Depending on the nature of the target, the potencies of the different boswellic acids varied between IC_50_ = 0.5 µM (βBA) and 4.1 µM (KBA) for the serine protease cathepsin G inhibition in cell-free assay, between IC_50_ = 3.0 µM (AKBA) and >10 µM (βBA and AβBA) for suppression of 5-LO activity in human neutrophils stimulated with A23187, and between 3 µM (AKBA) and 10 µM (KBA) for inhibition of mPGES-1 in cell-free assay [10,11]. An even stronger inhibition of mPGES-1 with IC_50_ = 0.4 µM were reported for 3α-acetoxy-8,24-dienetirucallic acid and 3α-acetoxy-7,24-dienetirucallic acid. mPGES-1 is an inducible enzyme that converts PGH_2_ formed by Cox-1/2 to the pro-inflammatory PGE_2_ [12]. Potent suppression of 5-LO in the range of IC_50_ = 2.9 µM and 5.1 µM was also observed for roburic acid, lupeolic acid as well as tirucallic acids [10]. These molecular targets were also confirmed in in vivo studies as molecular basis for the anti-inflammatory action of frankincense as perfectly summarized in the review of Efferth and Oesch [6].

In addition it was reported that 50 µM AKBA completely suppressed the nuclear transcription factor NF-κB in human myeloid KBM-5 cells, another key player in the development and progression of chronic inflammatory diseases [13]. Other boswellic acids lacking either the acetyl group (e.g., KBA) or the keto group (e.g., AβBA) were less potent and inhibited NF-κB only partially. Since the activation of NF-κB is a multi-step process, that involves the activation of the inhibitory κB kinase (IKK) complex, phosphorylation of inhibitors and their degradation, transport of NF-κB to the nucleus, binding to the NF-κB-consensus sequence, and activation of genes, the role of boswellic acids in this cascade was further investigated by Syrovets et al. [14]. They reported that pretreatment of transfected HEK293 cells with 10 µM AαBA and AKBA inhibited the NF-κB activity by 40.9 ± 9.8% and 76.9 ± 7.6%, respectively. Furthermore they demonstrated that AαBA and AKBA concentration-dependently (1–10 µM) inhibited IKK-mediated phosphorylation. Also the production of the pro-inflammatory cytokines TNF-α and IL-1β were inhibited by AαBA and AKBA in concentrations between 5–20 µM in human monocytes [15]. Previously Moussaieff et al. reported that incensole acetate also inhibited NF-κB activation, but this effect was observed at very high concentrations >100 µM [16].

With regard to the effect of frankincense on inflammatory and anti-inflammatory cytokines, Chervier et al. found out that a gum resin extract from *B. carterii* at a conc. of 10–200 µg/mL in sesame oil simultaneously inhibited the pro-inflammatory T helper (TH)-1 cytokines IL-2 and interferon (IFN)-γ, and promoted the anti-inflammatory TH-2 cytokines IL-4 and IL-10 in murine splenocytes [17].

Of course these pharmacologically active concentrations determined in vitro should be regarded with caution, as they can strongly vary depending on the assay conditions (e.g., species, cell-type, cell-free or cell-based assays, stimuli etc.) [18]. It was also reported that effects observed in cell assays cannot be reproduced under physiologically relevant conditions in the presence of albumin or in whole blood. Hence KBA efficiently suppressed 5-LO product formation in isolated neutrophils but failed to inhibit 5-LO product formation in human whole blood. This is not surprising, as boswellic acids being lipophilic acids are known to be substantially bound to albumin that is abundantly present in plasma (30–40 mg/mL) [18]. It must be also taken into consideration that the effects may vary depending on whether isolated purified phytoconstituents or the whole extract is tested. Nevertheless the abovementioned pharmacological active concentrations determined in common in vitro assays may serve as first indications for the efficacy of boswellic acids with regard to potential molecular drug-target interactions.

But the final pharmacological relevance of these data can only be correctly estimated when taking into consideration the oral bioavailability in human and the pathological role of individual molecular targets in different inflammatory diseases.

### 2.2. Oral Bioavailability in Human

The term bioavailability is used to indicate the fraction of an orally administered dose that reaches the systemic circulation as intact drug, taking into account both absorption and local metabolic degradation [19]. Often the terms “absorption” and “bioavailability” are erroneously used and considered interchangeable. However the absorption process represents only one of the steps involved in the passage of the drug from its site of administration into the systemic circulation [19].

Despite the widespread use of frankincense, only view preliminary pharmacokinetic studies were conducted. In order not to exceed the framework of this review, the focus was set on the pharmacokinetic studies carried out in at least six humans determining more than one boswellic acid. Two studies of Gerbeth et al. [20] and Sterk et al. [21] fulfilled these requirements, the results of which are summarized in Table 1. In the study of Gerbeth et al. the subjects included in a prospective, randomized, placebo-controlled double-blind pilot clinical trial on the effect of *Boswellia* on cerebral edema were asked to take the *Boswellia* medication at home [22]. No instructions were given regarding intake of medication or standardization of food. The blood samples were taken during weekly routine medical check-ups, irrespective of the time of medication intake [20]. The randomized, open, single-dose two-way cross over study of Sterk et al. was devoted to investigate the effect of concomitant standardized high-fat meal intake on the bioavailability of boswellic acids [21].

Compared to the high doses of *Boswellia* gum resin extracts that have been orally administered in the above studies, boswellic acids, especially KBA and AKBA, revealed very low bioavailability associated with a very high pharmacokinetic variability. The highest plasma levels could be determined for βBA that is also present at the highest concentration in the extract. Also at steady state the concentrations of the six boswellic acids determined after the oral administration of 4 × 786 mg/day for 10 days in one patient did not exceed 0.3, 0.1, 10.1, 2.4, 3.5 and 4.0 μM for KBA, AKBA, βBA, AβBA, αBA, and AαBA, respectively [23]. This might be attributed to the low aqueous solubility of boswellic acids, their high lipophilicity, gastrointestinal instability, low intestinal absorption, high accumulation within the enterocytes and intestinal metabolism by CYP enzymes as well as saturable kinetics [24]. These observations made in vivo are substantiated by low P_app_ values <3.00 × 10^−6^ cm/s determined for β-boswellic acids in the classical Caco-2 model using Hank’s balanced salt solution (HBSS) buffer without sink conditions [24]. However, when adapting the Caco-2 model to physiological conditions by the use of modified fasted state simulated intestinal fluid on the apical side and the addition of bovine serum albumin to the basolateral side in order to simulate sink conditions P_app_ values of 4.47 (βBA), 6.18 (AβBA), 5.52 (αBA) and 4.72 (AαBA) × 10^−6^ cm/s could be determined. These P_app_ values indicate moderate permeability according to Yee [25]. Also the P_app_ values of KBA and AKBA could be improved yielding P_app_ values of 29.54 and 17.83 × 10^−6^ cm/s, respectively, suggesting high permeability. The nevertheless low plasma concentrations of KBA in vivo can be explained by the extensive phase I metabolism observed for KBA and other non-acetylated boswellic acids (βBA and αBA) in human liver microsomes [26,27]. An explanation for the low plasma levels of AKBA might be the initially lower content of that boswellic acid in the extract combined with a greater distribution in different compartments [28]. The initial assumption that AKBA could be deacetylated to KBA following oral administration could not be verified [26].

Considering the pharmacologically active concentration in vitro, only βBA achieved sufficient high plasma concentration in the range of the IC50 values determined for serine protease cathepsin G and mPGES-1 inhibition, favoring a role of βBA as the most relevant anti-inflammatory boswellic acid [29]. At the same time pharmacological relevance of the putative targets of AKBA in vivo remains unclear. Hence most of the pharmacological effects of this boswellic acid, considered as the most potent one, were observed at relatively high concentrations in vitro, which revealed to be far above the plasma levels achieved after oral application of frankincense [30,31]. Unfortunately all pharmacokinetic studies focused on the determination of boswellic acids in plasma, so that no pharmacokinetic data are available for other potentially effective frankincense ingredients like roburic, lupeolic and tirucallic acids.

Based on that background it seems that bioavailability represents a major hurdle in the translation of the pharmacological potential of boswellic acids into therapeutic effects. Therefore several attempts were made to enhance the bioavailability of boswellic acids. As shown by Sterk et al. the bioavailability of boswellic acids could be significantly enhanced by concomitant intake of a fat-meal due to the solubilizing effect of bile acids [21]. Also the plasma concentration of KBA but not AKBA could be increased in 15 male volunteers who were administered a single dose of 800 mg *Boswellia* extract in fed conditions compared to the plasma levels obtained under fasted conditions. According to this study a significant but clinically infeasible dose increase of 10–15 fold the applied dose is required, to achieve pharmacologically relevant plasma concentrations for AKBA [32].

Further attempts to enhance the bioavailability focused on increasing the solubility of boswellic acids by developing new formulations based on the preparation of boswellic acid phosphatidyl choline complexes [33] and *Boswellia serrata* extract/phospholipid/pluronic f127 (1:1:1 *w/w/w*) formulations [34]. The latter formulation resulted in 26- and 14-fold higher plasma levels of KBA and AKBA, respectively, following the administration of 240 mg/kg to rats compared to the non-formulated extract. Also in the brain, 5-fold higher levels of AKBA compared with the non-formulated extract were determined eight hours after oral administration [34]. Casperome™, another formulation of *Boswellia serrata* extract and Phytosome^®^ (soy lecithin) at a 1:1 ratio revealed increased plasma levels and up to 35-fold higher concentrations of KBA and AKBA in the brain and 17-fold higher boswellic acid levels in poorly vascularized organs in rats [35]. Other formulations reported to lead to significant increase in the bioavailability of boswellic acids include Aflapin (composed of *Boswellia serrata* extract enriched in AKBA and non-volatile oil portion of *B. serrata* gum resin) [36], AKBA loaded poly-lactic-*co*-glycolic acid-nanoparticles [37], other nanotechnological formulations [38], as well as micellar delivery forms [39].

Obviously bioavailability seems to represent the major hurdle in the translation of the preclinical potential of frankincense extracts and boswellic acids into therapeutic effects [28]. Therefore it is worth to take a closer look at the efficacy of frankincense extracts in various preliminary human clinical trials that have been carried out.

### 2.3. Clinical Trials

A frequent problem of clinical trials carried out with traditional herbal remedies is their suboptimal or questionable quality, which hampers reliable statements on their clinical activity [6]. Therefore the focus will be set on randomized, double-blind, placebo-controlled clinical trials that used frankincense extract without other medications or other food supplements in diseases associated with inflammation, in order to securely trace back the observed effects to frankincense. An overview of these studies is given in Table 2. Unfortunately almost all clinical trials focused on clinical outcomes without determining inflammatory mediators. According to these studies clinically measurable improvements could be achieved with frankincense extracts enriched with AKBA in osteoarthritis [40,41,42,43,44]. Before Kimmatkar et al. reported decrease in knee pain, increased knee flexion and increased walking distance following the intake of 333 mg of *Boswellia serrata* oleogum resin with a minimum of 40% total boswellic acids three times a day for 8 weeks [45]. In contrast, a multi-centre controlled trial revealed no measurable effects of H15™ (3600 mg *Boswellia serrata* extract) in 37 outpatients with rheumatoid arthritis and chronic polyarthritis under constant therapy with steroids and anti-rheumatic drugs [46]. Although it may be assumed, that administration of H15™ to patients already treated with steroids would lead to additional effects, no further trials were conducted on patients with rheumatoid arthritis [15].

With regard to chronic inflammatory diseases no firm conclusion can be drawn regarding the efficacy of *Boswellia serrata* oleogum resin in the treatment of ulcerative colitis and chronic colitis because of the methodological weakness of the two studies conducted without blinding and randomization [47,48]. The greatest number of patients was included in the study of Gerhardt et al. [49] suggesting comparable efficacy of *Boswellia serrata* resin extract with mesalazine in Morbus Crohn. Another study with relatively small numbers of patients suggests promising effects of *Boswellia serrata* resin extract in the treatment of collagenous colitis [50]. But since no more studies were carried out since then, no valid data exist that support the use of frankincense extracts as monotherapy for chronic inflammatory bowel diseases [51].

Unfortunately only one study was carried out on the effect of *Boswellia serrata* extract in the treatment of asthma bronchiale, which however provided promising results [52]. Moreover Kirste et al. [22] confirmed previous observations made by Streffer at al. [53] as well as Boeker and Winking [54] regarding positive effects of concomitant administration of frankincense extract on cerebral edema associated with radiochemotherapy in patients with malignant glioma.

**Table 2:** Overview on the randomized, double-blind, placebo-controlled clinical trials addressing the efficacy of frankincense extracts in different diseases associated with inflammation.

Disease	Study Design	Dosage	Observations	References
Osteoarthritis knee	Pilot, randomized, double-blind, placebo-controlled on 48 newly diagnosed or untreated osteoarthritis patients	Self-administration of two tablets à 169.33 mg *Boswellia serrata* extract enriched in AKBA and βBA (AKBA 53.27 mg, βBA 20.83 mg, KBA 7.11 mg, AβBA 6.06 mg) for 120 days	↓ pain and stiffness ↑ motility of knee joints ↓ hs-CRP	[41]
Osteoarthritis knee	Pilot, randomized, double-blind, placebo-controlled on 60 patients with mild to moderate osteoarthritis	Aflapin 100 mg per day for 30 days (Aflapin contains *Boswellia serrata* extract enriched in AKBA with non-volatile oil of *Boswellia serrata*)	Clinically and statistically significant improvement in pain scores and physical function scores already after 5 days of treatment by ↓ 5-LO and ↓ TNFα	[42]
Osteoarthritis knee	randomized, double-blind, placebo-controlled on 60 patients with mild to moderate symptoms	Aflapin 100 mg per day compared to 100 mg 5-Loxin per day for 90 days	Clinically and statistically significant improvement in pain scores and physical functional scores 7 days after start of treatment ↓ TNFα induced cartilage degrading synovial fluid matrix metalloproteinase-3 and ↓ TNFα induced intercellular adhesion molecule (ICAM)-1 expression Aflapin better than 5-Loxin	[43]
Osteoarthritis knee	randomized, double-blind, placebo-controlled on 75 patients with mild to moderate symptoms	100 mg or 250 mg 5-Loxin (*Boswellia serrata* extract enriched with 30% AKBA) for 90 days	dose dependant clinically and statistically significant improvement in pain scores and physical functional scores 7 days after start of treatment ↓ TNFα induced synovial fluid matrix metalloproteinase-3	[44]
Osteoarthritis knee	randomized, double-blind, placebo-controlled on 30 patients	3 × 333 mg WokVel™ per day for 8 weeks (*Boswellia serrata* oleogum resin with a minimum of 65% organic acids or a minimum of 40% total boswellic acids)	↓ knee pain, ↑ knee flexion, ↑ walking distance and ability to climb stairs. After withdrawal of treatment symptoms returned	[45]
Morbus Crohn	randomized double-blind, verum-controlled parallel group on 83 patients		↓ Crohn’s Disease Activity Index (CDAI) by 90 in the H15 group and by 53 score points after therapy with mesalazine. Difference not statistically significant	[49]
Collagenous Colitis	randomized, double-blind, placebo-controlled multicenter trial on 25 patients	3 × 400 mg *Boswellia serrata* resin extract (H15™) per day for 6 weeks	Proportion of patients in clinical remission was higher in the *Boswellia* group compared to placebo group (63.6% vs. 26.7%). No significant difference in histology or quality of life	[50]
Bronchial asthma	double-blind, placebo-controlled on 40 patients	3 × 300 mg *Boswellia serrata* oleogum resin extract (S-compound™) per day for 6 weeks	Improvement of disease reflected in disappearance of physical symptoms and different signs as well as decrease in eosinophilic count in 70% of the *Boswellia* group compared to 27% of the placebo group.	[52]
Brain tumors	Prospective pilot, randomized, placebo-controlled double-blind study on 44 patients	3 × 4 × H15 (350 mg *Boswellia serrata extract)* starting with the first day of radiotherapy	reduction >75% of cerebral edema in 60% of the patients receiving *Boswellia* compared to 26% of patients in placebo group	[22]

↓ stands for decrease and ↑ stands for increase.

### 2.4. Conclusions

Definitely frankincense extracts are counted among the most studied herbal medicinal plants that are not authorized as herbal medicinal products. Nevertheless several outstanding issues remain that are essential for estimating the therapeutic efficacy. These issues relate to:

- possible role of boswellic acids with too high IC_50_ values that are not achieved in vivo in providing therapeutic effects
- pharmacokinetic properties of other promising frankincense ingredients i.e., tirucallic, lupeolic and roburic acid
- the influence of other extract ingredients on the pharmacological activity and efficacy
- effect of the pharmacological assays applied and experimental conditions on the outcoming results with regard to pharmacological activity and bioavailability
- the need for more well designed and high quality clinical trials to better underline positive/negative effects already observed

Alltogether *Boswellia serrata* reflects perfectly the general problems plant research suffers from. While a lot of data exist on possible molecular targets and pharmacologically relevant concentrations in vitro mostly for purified and isolated phytoconstituents, much less information is available on the pharmacokinetics of the tentative active ingredients of medicinal plants and even less information on clinical efficacy and the translation of in vitro data to the clinic. Therefore thought must be given to new methodological approaches that allow a better evaluation of the therapeutic potential of medicinal plants at a very early stage of research.

## 3. Curcumin: In Vitro IC_50_ Values, Bioavailability and Clinical Efficacy

**Figure 2:**
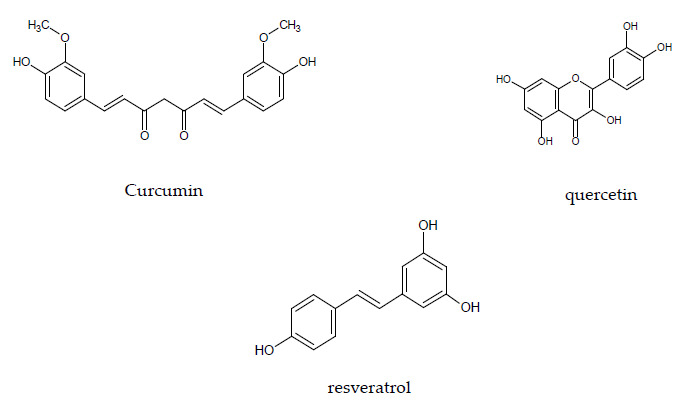
Chemical structures of curcumin, quercetin, and resveratrol.

Curcumin is a natural hydrophobic polyphenol [diferuloylmethane, (1*E*, 6*E*)-1,7-bis-(4-hydroxy-3-methoxyohenyl)-1,6-heptadiene-3,5-dione] that may be taken as an example for a well-studied isolated compound from medicinal plants (Figure 2). It is derived from turmeric (*Curcuma longa* Linn., Zingiberaceae) rhizome and represents the main component (~77%) of curcuminoid mixture along with demethoxycurcumin (~17%) and bisdemethoxycurcumin (~3%). In general curcuminoids in turmeric rhizomes range from 3% to 5% of dry weight and may vary depending on the geographical conditions [55].

### 3.1. In Vitro IC_50_ Values

Curcumin is a highly pleiotropic molecule that received substantial attention as an effective anti-inflammatory compound. Several mechanisms of action and numerous targets have been proposed that may rationalize its efficacy including enzymes, transcription factors, inflammatory mediators, and protein kinases [56,57].

Among the direct molecular targets of curcumin are COX-1 (IC_50_ 25–50 μM), COX-2 (IC_50_ > 50 μM), 5-LO (IC_50_ 0.7 μM), as well as mPGES-1 (IC_50_ 0.3 μM). The IC_50_ values for COX-1, COX-2 and 5-LO were determined in murine macrophage RAW264.7 and HT-29 colon cancer cells and that for mPGES-1 in microsomes of IL-1β stimulated A549 lung carcinoma cells. Also the suppression of cell signaling pathways at very high micromolar concentrations including NF-κB (IC_50_ 10–20 μM) through direct action on IkappaB kinase (IKK) in TNFα stimulated A549 cells has been reported [58]. Effects on TNFα and IL-1β in RAW 264.7 mouse macrophages stimulated with bacterial LPS were observed at concentrations of 100 μM curcumin [59].

Based on these data it seems reasonable to question the pharmacological relevance of those interactions that are characterized by low affinities as reflected by the high IC_50_ values determined in functional assays. On the other hand 5-LO and mPGES-1 may represent highly susceptible molecular targets because of the very low submicromolar IC_50_ values suggesting that these interactions may be easily achieved in vivo [60]. However pharmacokinetic studies revealed that curcumin is poorly bioavailable when administered orally. Either no curcumin was found at all or only low levels of curcumin metabolites were detected in serum or tissue [61]. Therefore it is worth to take a closer look at the pharmacokinetics of curcumin.

### 3.2. Oral Bioavailability in Human

In the majority of studies with crude curcumin powder at the dose of 3.6 g or lower curcumin was difficult to quantify despite very low limits of quantifications reported for the analytical methods applied reaching up to 0.0013 µM. Even at higher doses the concentrations of curcumin did not exceed 0.163 µM and that of total cumcumin (parent cucurmin + curcumin released from conjugated form by β-glucuronidase and sulfatase) did not exceed 0.271 µM in plasma when taken fasted or together with a regular diet. Only one study of Cheng et al. [62] revealed average curcumin peak serum concentrations of 0.51 +/− 0.11 µM, 0.63 +/− 0.06 µM and 1.77 +/− 1.87 µM following oral doses of 4, 6 and 8 g of crude curcumin powder. Probably premalignant lesions in the gastrointestinal mucosa of the included patients facilitated the absorption of curcumin. Extremely high total curcumin concentration of 8.686 µM and 5.70 µM were detected after the oral administration of 10 g and 12 g of curcumin formulated as Sabinsa C3 complex following a high fat meal (42% fat) [63].

In general the poor bioavailability may be attributed to several factors, namely low solubility (~11 ng/mL at pH 5.0), intestinal instability at pH < 3 and > 6, poor intestinal permeability (~0.07 × 10^−6^ cm/s in Caco-2 cells) and extensive first-pass intestinal and hepatic metabolism. The major metabolites formed were found to be predominantly curcumin mono-glucuronide along with curcumin sulfate and metabolites formed by the reduction of the allylic double bond, i.e., hexahydrocurcumin and tetrahydrocurcumin [55]. It seems that the enterocytes play a greater role as a site of curcumin glucuronidation than hepatocytes as reflected by a 2.5-fold greater total UDP-glucouronosyltransferase (UGT) activity in microsomes from the intestine compared to those from the liver [64]. All metabolites revealed very low pharmacological activity [63].

When administering curcumin together with 20 mg piperine, a known inhibitor of intestinal and hepatic glucuronidation, curumin bioavailability could be increased up to 20-fold [65].

A lot of approaches based on improving the solubility of curcumin resulted in a large number of curcumin formulations with enhanced bioavailability. A good overview of all the strategies applied is given in the review of Jamwal [66] and Adiwidjaja et al. [55] including formulations of curcumin-piperine complexes, solid lipid curcumin particles, curcumin fiber blends, curcumin essential oil mixtures, micronized curcumin, micellar curcumin, cyclodextrin inclusions, curcumin liposomes and curcumin phospholipid complexes. The best bioavailability enhancement reaching up to 185-fold improvement compared to the unformulated curcumin was achieved with NovaSol^®^ curcumin micelles composed of 7% curcumin powder and 93% Tween-80. Much smaller enhancement was reported for Biocurcumax™ (BCM-95^®^), a formulation of turmeric powder and essential oils leading to a 6.9-fold increase in bioavailability [66].

### 3.3. Clinical Efficacy

When entering the search terms “meta-analysis” and “curcumin” in combination 99 results are displayed in PubMed/MEDLINE for several systematic reviews/meta-analyses based on clinical trials that have been carried out for evaluating the clinical efficacy of curcumin in several diseases. The most important attributes of the meta-analyses as well as systematic reviews addressing the effect of curcumin on inflammatory diseases are summarized in Table 3. When several meta-analyses were found the two most recent ones were considered. In many studies the standard medication was continued in both the curcumin and placebo group.

The largest number of patients was included in randomized clinical trials (RCT) addressing the effect of curcumin on knee osteoarthritis [67,68]. In contrast only one pilot RCT carried out by Chandran et Goel [69] assessed the efficacy of curcumin in rheumatoid arthritis in the frame of a three-armed study comparing the effect of 500 mg curcumin with 50 mg diclofenac each taken two times a day and in combination for eight weeks. Interestingly the highest percentage of improvement in the disease activity scores was observed in the curcumin group. Also the RCTs addressing the effect of curcumin on ulcerative colitis and Crohn’s Disease resulted in overall positive outcomes [70]. In the case of ulcerative colitis it could be shown that the efficacy of adjuvant curcumin therapy may be improved by using enhanced bioavailable formulations and prolonging intervention times [71,72].

**Table 3:** An excerpt of recent meta-analyses and systematic reviews addressing the efficacy of curcumin in different diseases associated with inflammation.

Disease	No. of Clinical Trials and Patients Included in the Meta-Analysis/Systematic Review	Formulation	Observations	References
Primary knee osteoarthritis	Ten RCTs on 1287 participants	Formulations of turmeric or curcumin extract with increased bioavailability as adjunct or mono-therapy compared to placebo for up to maximal 8 months	↓ pain and ↑ function with reduced incidence of adverse events compared to NSAIDs	[67]
Osteoarthritis knee	16 RCTs on 1810 participants mostly from Asia	All forms of turmeric extracts compared to placebo or actives e.g., NSAID for up to 12 weeks (one study 16 weeks)	↓ pain and ↑ function compared to placebo and similar to NSAIDs with improved safety profile	[68]
Ulcerative colitis (UC) and Crohn’s disease (CD)	6 RCTs on a total of 374 patients with active mild to moderate UC and one RCT on 30 patients with mild to moderate CD	All forms of curcumin formulations compared to placebo for up to 6 months	Promising results. Two studies with low oral doses reported no significant differences and four with higher doses or better bioavailable curcumin reported significant reduction in clinical symptoms and higher remission rates	[70]

↓ stands for decrease and ↑ stands for increase.

However it is not known whether the positive effects of curcumin in ulcerative colitis result from its absorption and systemic bioavailability or from its local action on the intestinal mucosa [73], particularly since intestinal tissue concentrations of up to 13 µM were reported in humans [74]. Further studies on the efficacy of curcumin in bowel diseases are still ongoing [75].

Another meta-analysis and systematic review addressing the effect of curcumin-containing supplements on biomarkers of inflammation indicated a significant decrease in IL-6 and high-sensitivity C-reactive protein (hs-CRP) in patients suffering from metabolic syndrome [76]. The results also indicated that TNFα decreased, but this was not statistically significant. In addition one phase I trial confirmed the pharmacological relevance of mPGES-1 inhibition reporting a significant reduction in inducible PGE_2_ production in blood samples taken one hour after the administration of 3.6 g curcumin [77].

All in all most of the studies reported positive outcomes for curcumin with regard to the improvement of clinical symptoms in diseases associated with inflammation. Nevertheless there is still a limited breadth for high quality data to draw a final conclusion on the overall therapeutic utility of curcumin.

### 3.4. Conclusions

Whereas a lot of anti-inflammatory pharmacological targets have been proposed for curcumin, the functional link between the observed anti-inflammatory effects often remains unclear. In contrast to mPGES-1, representing a high-affinity target with IC_50_ values in the submicromolar range, other target interactions occur only at high curcumin concentrations, which are probably not pharmacologically relevant because they cannot be achieved in humans. This is attributed at first instance to the poor bioavailability of curcumin resulting from its low solubility, its poor permeability and high intestinal as well as hepatic metabolic rate. The main metabolites determined were curcumin glucuronide and sulfates in a ratio of 2:1 besides other reduced compounds. All of these metabolites were reported to be much less pharmacologically active than curcumin itself. Meanwhile a large number of curcumin supplements exist ensuring enhanced bioavailability associated with improved efficacy. Nevertheless promising anti-inflammatory effects were also reported for crude curcumin powder giving rise to the following questions regarding the:

- contrasting juxtaposition of achievable plasma concentrations and effective concentrations in vitro for the most targets identified
- possible accumulation of curcumin in plasma following repeated oral administration over long periods of time
- possible accumulation of curcumin in cells, which might lead to effective local concentrations
- possible activation of curcumin conjugates by intracellular glucoronidases or sulfatases

## 4. Quercetin and Resveratrol: Further Actives with a Lot of Unresolved Questions

Both quercetin (an abundant flavonoid found in a broad range of fruits and vegetables) and resveratrol (a polyphenol found mainly in peanuts, in the skin of red grapes and in red wine) share the same structural elements. They are nitrogen-free polyphenols with lipophilic moieties and more or less pronounced acidic character (Figure 2) [58]. Based on their polyphenol structure both substances are associated in first instance with anti-oxidative properties, but also numerous molecular targets have been identified for possible anti-inflammatory actions, often at high concentrations.

### 4.1. In Vitro Activity

In several in vitro studies quercetin proved inhibitory actions on a large number of targets including RaF/MEK1/ERK (IC_50_ 1–10 μM), NF-κB signaling ((IC_50_ 4–11 μM) and diverse kinases e.g., Janus kinase (JAK)3 (IC_50_ 2 μM) in THP-1 activated human monocytes and LPS-activated murine macrophages RAW246.7 leading to a reduction in transcription and expression of TNFα [58,78]. Moreover 25 μM quercetin blocked IL-1β, IL-6, IFN-γ, and TNFα secretion in human whole blood induced by LPS [79].

Similarly several potential anti-inflammatory mechanisms of action have been proposed for resveratrol including inhibition of 5-LO (IC_50_ 1–9 μM), COX-1 (IC_50_ 15–20 μM), 15-LO (IC_50_ 25 μM), mitogen-activated protein kinases (MAPK e.g., ERK, p38) (IC_50_ 25 μM), and NF-κB signaling ((IC_50_ 30 μM) [58].

### 4.2. Oral Bioavailability in Human

**Figure 3:**
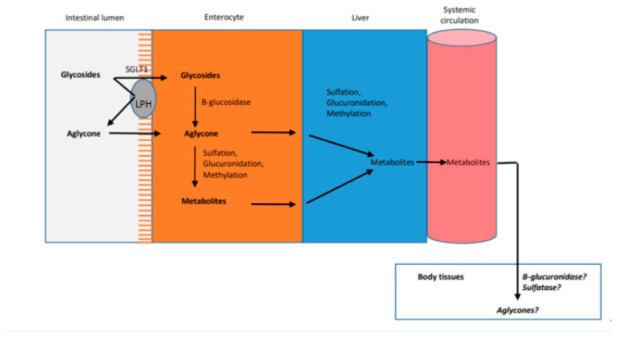
Overview on the absorption and metabolism of quercetin glycosides in the small intestine according to [80].

In general quercetin occurs in the plants in form of its glycoside with different attached types of sugars. Before they can be absorbed they are hydrolyzed by lactase-phlorizin hydrolase (LPH) enzyme to the aglycone, which is then passively absorbed into the enterocyte. Alternatively the glycosides can be actively transported by sodium-dependent glucose transporter (SGLT 1) into the enterocyte, where they are hydrolyzed by cytosolic β-glucosidase. The resulting aglycones then either passively diffuse into the hepatic portal vein or undergo phase I and II metabolism to produce glucuronidated, sulfo-substituted and methylated forms, which are transported via ABC transporters into the hepatic portal vein. The absorbed aglycones bound to serum albumin and the metabolites are transported to the liver. In the liver the remaining aglycones undergo phase I and II metabolism resulting also in the formation glucuronidated, sulfo-substituted and methylated forms, which are transported along with the intestinal metabolites into the systemic circulation for distribution to body tissues [80]. Available evidence indicated that quercetin glucoside is much better absorbed (3 to 17% in healthy individuals receiving 100 mg quercetin) compared to quercetin in the aglycone form lacking the attached sugar (2%) and other quercetin glycosides [78] (Figure 3).

The administration of 151 mg quercetin-3-glucoside capsule and 154 mg quercetin-4′-glucoside capsule resulted in similar peak plasma concentrations of 5.0 μM and 4.5 μM, respectively. A comparison of the absorption of quercetin from different food matrices (225 μmoles from onion, 325 μmoles from applesauce with peel, and 331 μmoles in form of rutin capsule) yielded the highest absorption rate of 0.74 µM for fried onions and 0.3 µM for the other quercetin sources. Moreover the accumulation of quercetin conjugates in plasma could be shown following the periodic ingestion of onion. Hence the concentration of glucuronide and sulfate metabolites increased from 0.04 μM to 0.63 μM after the consumption of 96.3 mg quercetin per day from onion slices over one week. The effective dose of quercetin supplement reported to have beneficial anti-inflammatory effect was found to be 500 mg [80]. Enhancement in quercetin aglycone absorption from the small intestine can be achieved by concomitant intake of dietary fat [80].

After resveratrol is orally ingested 77–80% is absorbed in the intestine [81]. Nevertheless extensive metabolism in the liver and in the intestine results in an oral bioavailability of considerably less than 1%. A phase I dose escalation study with single doses of 0.5, 1, 2.5 and 5 g yielded maximum plasma concentrations ranging between 0.3–2.4 μM for resveratrol. The major metabolites being resveratrol-3-*O*-sulfate and two monoglucuronides were detected at maximal concentrations of 3.7–14 μM and 0.9–4.3 μM, respectively [82]. Besides reduced dihydroresveratrol conjugates were also detected [83]. Also in another study with healthy humans taking 5 g resveratrol per day the most abundant circulating resveratrol metabolite, resveratrol-3-*O*-sulfate, reached considerably higher plasma levels (18 μM) compared with resveratrol (4 μM) [84]. Several attempts have been made to enhance the bioavailability of resveratrol by increasing solubility, preventing metabolism by inhibiting UDP-glucuronosyltransferases and sulfotransferases and developing nanoformulations. However all these approaches have not been extensively studied in humans but just on animal models [81].

### 4.3. Clinical Efficacy

Since quercetin and resveratrol are mainly used for their anti-oxidative effects, clinical studies addressing the anti-inflammatory properties are rare. Nevertheless it can be concluded from the clinical data available up till now, that the anti-inflammatory effects observed with quercetin in vitro could not be supported by studies in humans and generally remains unclear [78,80,85].

In case of resveratrol contrasting results were obtained. Hence a significant increase in TNFα was observed in ten healthy volunteers 24 h after the intake of a single dose of 5 g resveratrol [86]. In a different trial with nine healthy subjects who ingested 1g/day resveratrol capsules for 28 days a reduction in the plasma levels of TNFα together with a significant increase in the plasma antioxidant capacity was observed [87]. Also in a randomized double-blind and placebo-controlled study on 271 patients with takayasu arthritis taking 250 mg reseveratrol on a daily basis for three months, TNFα and other inflammatory markers like CRP were found to be stronger lowered in the resveratrol group compared to the placebo group [88]. Further positive effects of resveratrol were observed in the treatment of ulcerative colitis in the frame of a pilot study on 50 patients, in which a 500 mg dose for six weeks lead to the significant reduction in the plasma inflammatory markers TNFα, hsCRP and NF-κB as well as the colitis activity index [89]. Further TNFα suppression by resveratrol was observed in several other clinical conditions like chronic obstructive pulmonary disease (COPD), Type 2 Diabetes mellitus and CVD (cardiovascular disease) [90].

Then again another study based on the administration of 75 mg/day resveratrol for 12 weeks to non-obese postmenopausal women with normal glucose tolerance did not observe any change in inflammatory markers [91].

A possible reason for the contrasting results could be that low doses or a single but higher dose exerts no effect, whereas a moderate continuing intake seems be more effective [92].

### 4.4. Conclusions

All in all quercetin and resveratrol are further examples of well-studied substances in vitro that are poorly bioavailable because of extensive metabolism to glucuronidated or sulfated metabolites. Interestingly the plasma concentrations of the metabolites exceed that of the parent compound several folds, giving rise to the question of:

- the role of quercetin and resveratrol conjugates as well as the respective parent compounds in the mediation of the pharmacological effects
- possible activation of metabolites by glucuronidases and/or sulfatases in the tissues
- achievable tissue concentrations of the active substances

**Figure 4:**
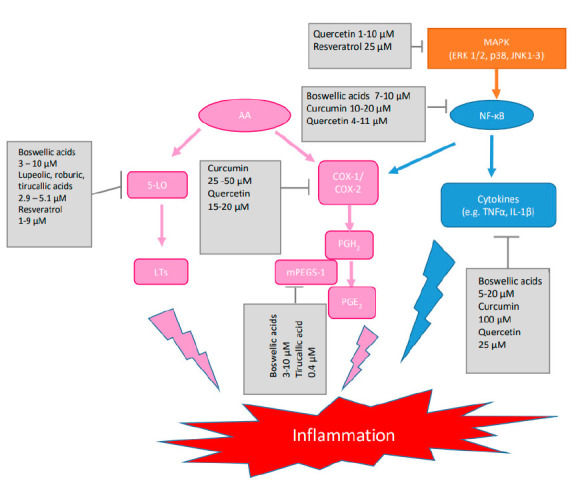
Simplified excerpt of the relation between different inflammatory mediators visualizing the point of attack of boswellic acids, curcumin, quercetin and resveratrol and the corresponding IC_50_ values. AA = arachidonic acid, COX-1,-2 = cyclooxygenase-1, -2, ERK 1/2 = extracellular signal-regulated kinase 1/2, IL-1β = interleukin 1β, JNK1-3 = c-Jun-N-terminal kinase 1-3, 5-LO = 5-lipoxygenase, LT = leukotrienes, MAPK = mitogen activated protein kinase, mPGES-1 = microsomal prostaglandin E synthase-1, NF-κB = nuclear factor ‘kappa-light-chain-enhancer of activated B-cells, PGE_2_ = prostaglandin E_2_, PGH_2_ = prostaglandin H_2_, TNFα = tumor necrosis factor α.

For a better overview a rough simplified excerpt of the relations between different inflammatory mediators visualizing the respective points of attack and the corresponding IC_50_ values for the here mentioned exemplary anti-inflammatory herbal components is shown in Figure 4.

## 5. Is Tissue Distribution a Better Surrogate Than Plasma Levels?

All the above examples have in common that the achieved plasma concentrations of the tentative active ingredients were found to be far below the IC_50_ values (the concentration of an inhibitor where the response of binding is reduced by half) determined for most of the putative pharmacological targets in vitro. Moreover these substances as well as their conjugates are highly bound to plasma proteins, reducing further the free unbound drug concentration [93]. At the same time promising clinical effects have been reported in several clinical trials despite the poor pharmacokinetic behaviors for these substances.

According to our traditional understanding of pharmacokinetics, it is assumed that pharmacological activities of drugs are closely related with the respective free unbound drug concentration in plasma [5]. Excellent herbal substances therefore should yield high free drug plasma concentrations to achieve pharmacological effects. However the demonstrated therapeutic effects indicate a pharmacokinetic behavior of herbal substances that is obviously not the same as what we expect in our conventional theory. Therefore we have to rethink about our pharmacokinetic understanding and the real factors that influence pharmacological activity of herbal substances in vivo, in order to unlock the “black box” of herbal substances.

### 5.1. What We Know about the Tissue Distribution of Boswellic Acids, Curcumin, Quercetin and Resveratrol

In the blood stream, drugs are transported partly in solution as free (unbound) drug and partly reversibly bound to blood components (e.g., blood proteins, blood cells). The extent of drug distribution into tissues after entering the systemic circulation depends on the degree of plasma protein and tissue binding. For all the mentioned compounds protein binding dominates leading to a small apparent volume of distribution (V_d_). This is reflected for example by V_d_ values of 1.8 L/kg or 3.7 L/m^2^ determined for resveratrol and quercetin, respectively [83,94]. Highest tissue levels of quercetin were found in the lungs, followed by the kidney, liver and heart corresponding to 26%, 13%, 11% and 10% of the plasma concentration following the oral administration of 12 mg/kg/d quercetin aglycone over a period of eight days to rats. The administration of isoquercetin (glucosylated quercetin) increased the respective quercetin tissue concentration percentages to 27%, 39%, 11%, and 25%, respectively [95]. The highest concentrations of resveratrol were detected in the liver and kidney and trace amounts in the brain, lungs, heart and spleen [96]. In contrast curcumin was detected in brain in a comparable concentration like that determined in kidney and liver following the administration of 20 mg/kg curcumin by tail vein injection to mice. This is reflected by a higher V_d_ of 9.1 L/kg [97]. Interestingly higher tissue to plasma ratios (>10) were determined for curcumin glucuronide compared to curcumin (<10) four hours after the i.v. administration of 20 mg/kg curcumin via the lateral tail vein to rats [98]. Administration of 40 mg/kg of the more lipophilic prodrug, curcumin diethyl succinate, resulted in a better distribution of curcumin and curcumin glucuronide in major internal organs [98]. Mice chronically fed with 2.5–10 mg/day curcumin for four months revealed brain concentrations of 1.35 µM [99]. In general the detected brain concentration of curcumin were lower following oral administration compared to intraperitoneal or intravenous injections. Nevertheless, these studies clearly demonstrate the ability of curcumin to pass the blood brain barrier (BBB) despite its poor oral bioavailability, which is attributed to its lipophilicity [100].Boswellic acids could also be detected in the rat brain eight hours after oral administration of 240 mg/kg *Boswellia serrata* extract to rats with the highest concentration determined for βBA corresponding to 2.33 μM, falling thus in the range of pharmacologically active concentrations. Compared to KBA and AKBA with a brain-to-plasma ratio of 0.2 and 0.4, respectively, βBA yielded a brain-to-plasma ratio of 1, indicating facilitated blood-brain permeability due to its greater lipophilicity [27]. By enhancing the solubility of boswellic acids the brain availability could be further improved for all boswellic acids reaching up to five times higher levels for βBA, which was also accompanied with a corresponding increase in the plasma levels [34].

### 5.2. The Role of Protein-Facilitated Uptake into Tissues

The widely accepted free drug theory (FDT) explains that plasma protein binding is a rapid equilibrium process allowing a constant concentration of free drug, and in the absence of energy-dependent process, this free drug concentration in plasma is the same in tissues and extracellular fluids at steady state. The other main principle of the free drug concentration theory is that only free drug can reach the site of action, and therefore the free drug concentration is what drives the pharmacological effect of a substance [101].

Meanwhile it seems to be more and more recognized, that the drug concentration taken by the cells in the presence of extracellular binding proteins could be substantially higher than expected based solely on the free drug concentration. This additional increased uptake of highly bound drugs might be driven by the so-called albumin-facilitated or protein-facilitated uptake [102]. The exact mechanism by which these binding proteins may facilitate the uptake of a drug in cells is still unknown. Some of the hypotheses relate the facilitated uptake to the presence of ionic attractions between the protein-drug complex and the cell surface, which may result in a marked reduction in the diffusional distance of the extracellular protein-drug complex. It is also believed that conformational changes in the albumin-drug complex attributed to interactions of the albumin with membranes in an acidic microenvironment may reduce the albumin binding affinity of the drug. Another theory takes the role of uptake transporters into account [102,103,104]. Hence high affinity binding of drugs to cell membrane proteins such as organic anion transporter proteins (OATPs) may be able to change the equilibrium of the nonspecific binding between drugs and plasma proteins, i.e., if a highly protein bound drug has a higher affinity for a transporter than albumin, the transporter may be able to strip the drug from the protein before the drug dissociates itself and is at binding equilibrium. In general the protein-facilitated uptake mechanism has been found to occur in liver/hepatocytes, myocytes, adipocytes, proximal tubules, perfused kidney, brain and human embryonic kidney cells overexpressing OATP1B1 and 1B3 [101]. All in all this would mean that protein binding is not generally restricting the access of these compounds to tissues.

In this sense binding of the poorly water-soluble resveratrol to plasma proteins like lipoproteins, hemoglobin, and HSA is essential for its body distribution and bioavailability. It could be shown that besides a passive diffusion influx, resveratrol enters the liver cells by an active process involving OATPs that bind the resveratrol-albumin complex and deliver resveratrol in a similar way to the fatty acid uptake [105].

There are also indications from commonly used synthetic drugs, such as benzodiazepines, and steroids that demonstrate higher concentrations in the CNS, than their unbound fraction would suggest [106]. In case of diazepam for example the fraction in the CNS was eight times higher than the in vitro determined unbound fraction. This proves that compounds having a high protein binding may nevertheless readily dissociate and enter the CNS [107]. Meanwhile the availability of high-throughput equilibrium dialysis or brain slice methods used to estimate the unbound fraction of drugs in brain highlighted that the in vivo blood brain barrier permeability (i.e., the rate of drug delivery to the CNS) is influenced by their ability to bind to brain tissue [108].

Translating these observations to plant research, this would mean that in contrast with the previous assumption, that protein binding might reduce the free unbound pharmacologically active form, binding to albumin reveals to be essential for the delivery of the bound form at the cell surface, facilitating consequently membrane uptake to finally produce its biological effect [93]. Therefore much more attention should be drawn to the drug concentration at the site of action.

### 5.3. Role of Tissue β-Glucuronidases and Sulfatases

Another important issue that should be taken into consideration when explaining the therapeutic effects of herbal substances with unfavorable plasma levels is the role of β-glucuronidases and sulfatases localized in tissues. They might release active herbal substances from its glucuronic acid and sulfate conjugates, promoting thus their pharmacological activity in tissues.

This hypothesis may be underlined by the results of a distribution study following the oral administration of ^3^H-labelled resveratrol to rats via gastric gavage [96]. Hence the predominance of conjugated resveratrol in plasma 2 h following gastric gavage would suggest that these conjugates are also most likely to reach the tissues. However only the liver and kidney retained the conjugate form, whereas in other tissues such as lung and brain, detectable levels of the aglycone were found. Moreover the tissues including the liver retained a higher ratio of the aglycone to the glucuronide at 18 h post-gavage compared with their ratio at 2 h. Due to the absence of the aglycone in the plasma, the results support the possibility, that the ubiquitously existing β-glucuronidase could convert the conjugated form back to resveratrol. In another study on the distribution of resveratrol and its metabolites following the administration of 10 mg/kg resveratrol per oral gavage to mice also much higher resveratrol to metabolite ratios were observed in the lung, heart, thymus, and muscle. In line with the previous study no resveratrol conjugates could be detected in brain [109].

Similar observations were made with curcumin. Following the administration of curcumin to mice curcumin glucuronide was the primary metabolite detected in serum accounting for 99.7% of total curcumin. In contrast the aglycone curcumin was the major metabolite detected in bones accounting for 69.4% of total curcumin. Hence the aglycone concentration in bones was 6.4-fold higher than in the circulation. This might be attributed to the high deglucuronidation activity known for bone marrow cells [110].

Inflammation might even increase the glucuronidase deconjugation activity. This was shown in mouse plasma after the i.v. injection of lipopolysaccharide (LPS), where the β-glucuronidase activity increased with time as did the levels of inflammation marker, TNFα, and soluble intercellular adhesion molecule-1. In agreement with this the peak of luteolin monoglucuronide decreased to about half and the ratio of luteolin to luteolin monoglucuronide increased in LPS treated rats [111].

Quercetin glucuronides were also shown to be deconjugated in vitro in cultured macrophages and in homogenates from human liver and small intestine [112]. Furthermore relative high concentrations of quercetin in its free form were detected in several tissues in pigs despite it being undetectable in plasma [113]. An increased deconjugation activity into the active quercetin aglycone upon inflammatory activation by LPS was also reported for quercetin glucuronide that was bound to the cell surface proteins of peritoneal mice macrophages. This was attributed to the LPS induced extracellular acidification, which is required for the activity of β-glucuronidase [114]. In other in vitro experiments carried out in homogenates from the mesenteric bed of rats a clear correlation between pharmacological activity of the aglycone and deconjugation of quercetin glucuronide could be demonstrated [115,116].

All together these examples show, that although the glucuronide and sulfate conjugates are in general less active, they may be easily converted back to the non-conjugated forms, acting thus as a depot for drugs in free form within target tissues. Hence the pharmacological activity of herbal substances is also linked to the kinetics of the conjugates, the reason why studying the pharmacokinetic behavior of the conjugates should receive more attention in the future.

### 5.4. Further Examples for the Relevance of Tissue Concentration

For other drugs the concentration in tissue may also provide better correlation to therapeutic effects than plasma concentrations. This is especially true for locally acting compounds in the skin as e.g., melatonin and secosteroids.

Besides being a hormone controlling the sleep-wake cycle in human, melatonin has been identified in about 120 plant species in variable concentrations. Of particular interest are its cutaneous pleiotropic effects as a consequence of its local concentration and metabolism occurring directly in the skin at the site of action [117]. Circulating and orally administered melatonin is predominantly metabolized in the liver through O-demethylation and 6-hydroxylation in order to be conjugated and excreted as glucuronides and sulphates. This first pass effect is the main reason for its very low bioavailability following oral administration. In contrast its metabolism in human skin is targeted at producing active metabolites including the formation of 2- and 6-hydroxymelatonin, two methoxykynuramine, one methoxytryptophane and one methoxytryptophol derivatives. These metabolites together with melatonin are responsible for the protective effects against UV solar skin damage or other oxidative stress factors caused by inflammation or ionizing radiation through direct radical scavenging and antioxidative enzyme stimulating action. By this way UV mediated damaging events such as lipid peroxidation, protein oxidation, mitochondrial and DNA damage may be reduced. For supporting this complex intracutaneous melatoninergic antioxidative system (MAS) in clinical dermatology exogenous melatonin should be applied topically rather than orally, as orally administered melatonin appears at rather low levels in the blood due to prominent first-pass degradation in the liver, which limits skin access [117]. For meeting this requirement of local high intracellular concentration, the concentration of melatonin and its even more active metabolites should be directly determined in the skin, i.e., in the tissue not in plasma [118].

The same holds also for the hydroxymetabolites of the secosteroids vitamin D3 (D3) and lumisterol (L3), which like melatonin and melatonin metabolites also protect the skin from or reverse UV-B induced damage. This is achieved by downregulating pro-inflammatory responses via suppression of NFκB activity and its downstream cytokines including TNFα and INF-γ, inhibition of IL-17 production, and stimulation of keratinocyte differentiation [119]. As in case of melatonin the active hydroxymetabolites are also formed locally in the skin due to the presence of the respective enzymes like e.g., the mitochondrial CYP11A1 in the skin [120]. Again it is the tissue concentration that should be monitored, not the plasma concentration because its rather the subcellular localization of secosteroid hydroxymetabolites that effect the ability to interact with various regulatory proteins for exerting its protective effect against various stress factors.

### 5.5. Methods to Determine the Tissue-to-Plasma Ratio

The determination of the tissue-to-plasma ratio therefore represents a much more conclusive surrogate for the pharmacological activity of herbal substances than the plasma levels. In fact, the importance of tissue binding cannot be neglected, as 98% of all proteins likely to contain binding sites for drugs are located extravascularly. However the difficulties inherent to studying the interactions with the proteins of fixed tissues hinders the readiness to carry out these essential investigations in contrast to the predestined plasma pharmacokinetics.

#### Distribution Dialysis

Apart from tissue distribution studies carried out in vivo in animals the simplest way to simulate binding competitions between tissue components and plasma proteins is a simple in vitro technique named distribution dialysis that has been established long time ago [121,122]. In general equilibrium dialysis makes use of two side-by-side chambers that are separated by a dialysis membrane. One chamber contains a buffer and the other diluted plasma or tissue with the drug. Once diffusion equilibrium is reached the free and bound drug concentrations can be determined. The advantage of tissue distribution dialysis is that both dialysis chambers contain a binder, and the drug is allowed to distribute between the two different binding systems. Hence the drug is dissolved in diluted whole blood (ten percent) contained in one dialysis chamber and dialyzed until equilibrium against homogenized tissue (10%). The distribution is then expressed as drug concentration ratio of the two chambers, e.g., the tissue-to-plasma ratio. This ratio is found to be below unity for acidic drugs, indicating the predominance of plasma binding and low partitioning into membranes and neutral lipids. Basic drugs on the other hand are characterized by a tissue-to-plasma ratio far above unity being for most tissues around 10. Interestingly the variations in the fraction bound among the various tissue homogenates for ten model drugs were found to be very small, suggesting uniform non-specific tissue binding. In general this model may be used as alternative to distribution studies in animals for neutral and acidic drugs because of its very good correlations with in vivo data. Only the distribution for basic drugs is underestimated for the liver, lungs and kidneys but not for brain, muscles and adipose tissues. This may be explained by the preferential accumulation of basic drugs in lysosomes and their disruption upon homogenization or prolonged incubation time. However this disruption is not a problem for tissues with less abundant lysosomes as brain, muscle and adipose tissue or for acidic lipophilic drugs in all tissues. A way for measuring true tissue binding for lipophilic drugs in tissues rich in lysosomes would be the use of preparations such as viable cells that maintain structural integrity [122].

Over the years other techniques were utilized in vivo to gain insight into the pharmacokinetics at the site of action and estimating drug exposures in different compartments. A good overview on the individual techniques that may be applied for the different tissues, their advantages and disadvantages is given in the review of Rizk et al. [123]. The most common applied techniques are microdialysis and positron emission tomography (PET) imaging with radiolabeled drugs. Microdialysis has emerged as very informative, albeit invasive, method for continuously measuring tissue concentration. In contrast to the invasive microdialysis technique PET scanning is non-invasive and able to collect multiple images following a radiolabeled dose to determine drug concentrations in tissues over time. However it is expensive and resolution is not always sufficient. Moreover the PET label may impact the drug distribution and the signal may represent the parent drug or its metabolite [123,124,125,126,127,128,129,130].

**Figure 5:**
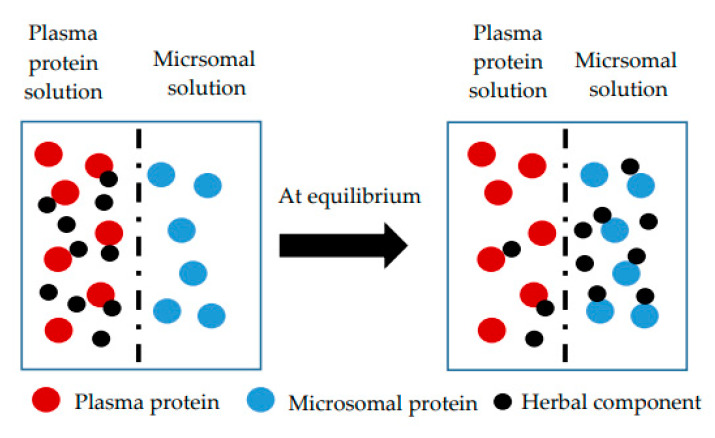
Schematic representation of a competitive dialysis approach making use of plasma-microsomal protein co-incubation according to [123].

All the above analysis techniques share the disadvantage of requiring access to organs and tissues and therefore usually to animal studies. Therefore more and more prediction methods for estimating tissue to plasma partition coefficients for individual drugs are used; some of them requiring an in vivo component and others use more readily available in vitro inputs. Recently the prediction of the tissue to plasma partition coefficient could be facilitated by developing a prediction method based on determining the microsomal partitioning and making use of a single equation for neutral drugs, acids and bases [126]. In other prediction methods log *P* is used to model phospholipid partitioning and several equations are necessary to model neutral, acidic and basic drugs. A shortcoming of using log *P* calculated from the octanol:water partition coefficient (*P*) to represent phospholipid partitioning is the lack of orientation specific interactions with phospholipid membranes resulting in mechanistically unsound assumptions. By contrast depending on partitioning into microsomes (unsorted phospholipid vesicles) represents a great benefit, as it allows measuring interactions with all phospholipids for both charged and uncharged species in simple experimental settings leading to more accurate predictions (Figure 5) [124].

Unfortunately prediction models are difficult to apply for herbal substances that are normally available in the form of extracts as they cannot reflect the interactions of the different extract ingredients. Therefore there is still a great demand for alternative experimental designs that allow conducting equilibrium dialysis studies in vitro ensuring at the same time a proper assessment of the free tissue to plasma ratio for herbal substances without the need for organs or tissues. The competitive dialysis approach as presented in Figure 4 making use of plasma-microsomal protein co-incubation may represent such a promising alternative experimental setting [125]. The experiments may be also conducted with single use rapid equilibrium dialysis (RED) plates, that are easy to use, provide reasonable throughput and are amenable to automation. Last but not least they are above all resource-sparing alternatives for organs and tissues required to determine tissue binding. The limitations of quantifying low levels of compounds that are extensively bound to plasma may be overcome by modifying the experimental system, e.g., applying longer incubation times or diluting the plasma. Even though this approach has only been proven on limited number of model drugs up till now, the results obtained suggest that it may be an appropriate model for estimating the impact of protein binding on clearance prediction. Moreover its application may extend beyond in vitro in vivo correlation of clearance to evaluate other parameters such as species differences in protein binding and free tissue to plasma ratios [125].

### 5.6. Conclusions

The above examples show how critical the correct estimation of tissue concentration may play in the evaluation of pharmacological activity. Hence drug exposure at the site of action may not be in equilibrium with the blood levels, limiting the utility of blood sampling as the only surrogate for pharmacological activity. At the same time the thoughts described manifests our great deficit in understanding the tissue accumulation of pharmacologically promising herbal substances. Therefore studies devoted to determining the drug concentration at cellular sites should receive much more attention in the future in order to be able to better estimate possible accumulations of drugs up to pharmacologically active concentrations in target organs.

## 6. The Challenges of Complex Multi-Component Herbal Mixtures

Whereas the previously mentioned pharmacokinetic aspects may be more easily considered in case of single herbal substances, they soon reach their limit with complex multi-component herbal mixtures. Therefore previous efforts to identify pharmacologically active compounds in herbal medicines have focused on the screening of isolated single compounds. But it has to be taken into consideration that the level of single compounds contained in herbal medicines is generally too low to exert sufficient pharmacological effects against complex diseases. Consequently such screening strategies are unable to uncover the combinatory compounds contributing to the holistic effects of herbal medicines. This represents a major bottleneck in providing sound evidence for supporting the clinical benefits of herbal medicines [126]. Given knowledge of this major obstacle to understanding herbal medicines recent research, especially in China, was devoted more and more to identify the exact composition of combinatorial components as well as their metabolism accounting for the whole efficacy of herbal medicines.

### 6.1. Identification of Combinatory Compounds Attributing to Pharmacological Activity

In fact identifying those compounds that act in a synergistic or additive mode is a key step to resolve the multiple-compound and multiple-target holistic effects of herbal medicines. Such an identification procedure often begins with chemical profiling of the constituents of herbal mixtures, followed by fractionation on semi-preparative columns, and testing the fractions and/or isolated compounds in comparison to the whole mixture in biological assays in vitro [126,127]. In the first step of chemical profiling the individual components are commonly separated by HPLC-UV prior to structural characterization with Q-TOF MS generating total ion chromatograms in the positive and negative mode. The detected compounds may be identified by comparison with available reference compounds or literature information. For focusing on the most important components the normalized peak area ratio (%) may be applied as selection criteria. Hence selection thresholds may be defined as 10%, 1% or 0.1% including different numbers of peaks and related components accordingly. The selected candidates in light of the defined threshold may be then isolated at the milligram levels by semi-preparative HPLC. Next the combinatorial components covered by a selected threshold are tested in different in vitro cell systems in comparison to the total herbal mixture. For comparing the efficacy of the combinatorial candidates with the total herbal mixture the concept of bioactive equivalence is applied that is similar to bioequivalence in pharmacokinetics, i.e., the ratios of the efficacies of the selected candidates should fall within a given range of the total herbal mixture for a certain assay. Depending on the resulting efficacy assessment selection criteria may be stepwise adjusted until bioactive equivalence with the total herbal mixture is achieved. As long as the requirements for activity in the range of 70–143% of the reference total herbal mixture are not met, bioequivalence studies with the components belonging to the following selected threshold will be carried out, until the fraction with bioequivalent activity to the total herbal mixture in different cell systems is identified. This approach is based on the assumption that not all components contribute to the efficacy of herbal medicines, and some constituents contribute largely to the effects. Other reference parameters may be a common chemical structure, or binding affinity to bioactive macromolecules. Finally the results may be verified in rat studies [127].

All in all such a process is extremely time-consuming, labor intensive and poorly efficient. A much more convenient and efficient method to identify potential pharmacologically active components in complex mixtures is the affinity-based screening assay [130]. In fact bioanalytical screening techniques have been proposed as advanced alternatives to the classical bioassay-guided fractionation for active compound identification. Various types of targets can be utilized for affinity-based assays including enzymes, receptors, neurotransmitters, transport proteins, DNA, and any other bio-macromolecules, even cell membranes and living cells. Among diverse target immobilization methods ligand fishing has emerged as a convenient and efficient technique to fish out potential ligands from complex mixtures. In ligand fishing experiments, any compound with affinity to the immobilized target will be remained for further analysis while non-binding compounds will remain in the extract and will be discarded. The high-selectivity and high-throughput of ligand fishing assays may be further supported by specific enrichment steps, that may be applied when separating the ligand-bound active components from the rest of the mixture using for example centrifugation, ultrafiltration, equilibrium dialysis, microdialysis, magnetic beads, and affinity chromatography. The following analysis steps are generally performed by HPLC or mass spectrometry. Depending on whether the separation and analysis of ligand-binding components occurs simultaneously or one after the other the approaches may be classified into on-line or off-line mode. For on-line mode the incubated sample solution is directly analyzed without further enrichment steps by chromatographic approaches and the active ingredients from the mixture can be tentatively determined by comparison with the chromatograms of the original sample. A detailed overview on different ligand fishing approaches and the successful application of this strategy is given in the review of Zhuo et al. [128].

Various computational approaches particularly structure-based virtual screenings may also be used for preselecting potential pharmacologically active herbal ingredients. This approach was applied by Wang et al. who succeeded in identifying four TCM herbs highly enriched in active ingredients that bind to a nucleoprotein of Ebola virus. For proof of principle the respective extracts were incubated with the nucleoprotein followed by separation of the protein bound compounds and identification of the binding herbal active ingredients via LC-MS/MS using high-resolution mass spectrometry. By applying multi-variate analysis for the incubation solutions, herbal extracts and control solutions the preliminary selected four TCMs could be reduced to three that are found to be the most active [129].

Consequently, the increasing chemical and pharmacological possibilities of identifying combinatorial components in complex herbal mixtures should encourage us to focus more and more on a “network” approach, in which multiple compounds interact in vivo with multiple targets with interdependent activities to achieve an optimal effect. At the same time the quality standards in the field of ethnopharmcological field studies and phytopharmacology as addressed by Heinrich et al. should be followed, in order to assure a high level of reproducible evidence-based medicinal plant research [130,131].

### 6.2. Pharmacokinetics of Multicomponent Complex Herbal Mixtures

It is well known that the pharmacokinetics of a single compound may be significantly different from the same compound in a multi-component mixture. But due to the complexity of both herbal medicinal plants and biological samples (blood, urine, tissues) and the associated technical challenges quantitative measurements of time-dependent concentration profiles of bioavailable multiple components exceed the scope of commonly applied traditional research. This has led to significant limitations in understanding the efficacy of herbal medicines. In fact it is technically challenging to identify each metabolite and to assess the human response to exogenous substances in a complex network involving a large number of variables. Thus the majority of absorbed herbal components and their metabolites are often intermixed against the background of endogenous substances in biological samples. But recent advances in liquid and gas chromatography as well as capillary chromatography coupled with mass spectrometry (LC-MS, GC-MS, and CE-MS) and nuclear magnetic resonance (NMR) made it possible to simultaneously detect various multiple components and their metabolites [132].

These are important prerequisites for metabolomic profiling techniques that have already clearly demonstrated their value in elucidating the interaction of the biological system’s genome with its environment and have been recently applied in the pharmacokinetic analysis of drugs, xenobiotics and several nutrients [133]. In fact the application of LC-MS based metabolomics profiling has increased exponentially over the last decades as it offers a novel way to simultaneously measure hundreds and thousands exogenous components from herbal medicines and to differentiate them from endogenous substances. The main difference between LC-MS based metabolomics and classical LC-MS is the data processing procedure to differentiate exogenous substances and their metabolites from the endogenous background. The classical approach utilizes arbitrary endogenous background subtraction and/or knowledge-based mass change filtering, which takes advantage of known mass differences between the parent ion and metabolite ions. For metabolomics approaches multivariate data processing methods (e.g., deconvolution, alignment, integration) coupled with multivariate analysis (e.g., principle component analysis (PCA), partial least squares discriminant analysis (PLS-DA), hierarchical clustering analysis (HCA) and Bonferroni correction filters are employed. This strategy may be also applied for untargeted metabolomics i.e., screening bioavailable herbal components and their metabolites with no prior knowledge of the chemical composition of the herbal mixture [134,135]. Because of its high sensitivity, specificity and reproducibility liquid high-resolution mass spectrometry is especially suited for untargeted metabolomics. In the frame of comparative analyses user-defined values for statistical significance represented by respective *P*-values, and fold changes greater than two qualify potential unknown bioavailable components and their metabolites for further testing and identification. For these purposes numerous commercial and freely usable data-processing packages are available as well as multiple databases that are continuously improved [136].

Compared to the classical approaches metabolomic strategies have clear advantages, firstly in the capacity of handling a great number of variables allowing a shift from targeting analysis to profiling the complete set of exogenous substances and metabolites, and secondly, in the unbiased selection of variables that are significantly altered, which facilitates the discrimination between exogenous and endogenous substances. Moreover it is very useful in identifying significant bioavailable herbal components and their metabolites paving the way for the identification of pharmacologically active components of herbal medicines that have been not yet extensively studied. In fact several publications reported the use of metabolomics to capture both drug-derived and drug-induced metabolites [132]. Due to these advantages new methodological approaches become achievable that may start also with mapping absorbed substances and metabolites in vivo followed by in vitro characterization. This is in contrast to the commonly applied knowledge based methodology that starts with in vitro identification followed by in vivo characterization [134].

**Figure 6:**
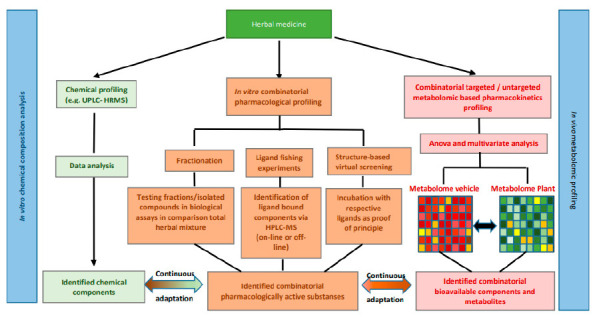
An exemplary overview on a possible approach for conducting combinatorial medicinal plant research.

The major objectives of applying metabolomics-based pharmacokinetic studies are to qualitatively identify and characterize the bioavailable herbal components “what is absorbed” and their metabolites “what is produced” and present their semi-quantitative time courses in the global subject metabolite pool following the administration of herbal medicines. An example for the successful application of phytochemical and metabolomic profiling coupled with multivariate statistical analysis is the simultaneous pharmacokinetic monitoring of several components of Huangqi decoction (HQD, consisting of *Radix Astragali* and *Radix Glycyrrhizae*) and the metabolic responses in healthy Chinese volunteers [137]. In order to reduce sources of variation and to assure reproducibility of the experimental results, it is preferable to have a detailed phytochemical characterization of the medicinal plant or the extract applied. Conversely it is important to define the lead substances that should be monitored for quality control of medicinal plants on the bases of the insights gained from metabolomics and combinatorial pharmacological studies. An overview on a possible approach for conducting combinatorial medicinal plant research is given in Figure 6.

In addition metabolomic approaches may be also utilized as an important tool for quality control of herbal medicines capturing differences in the contents of active ingredients due to growing areas, different cultivars, preparation, and adulteration. Furthermore metabolomics may be also successfully applied to determine the metabolic pathways and molecular targets helping to clarify the efficacy of herbal medicines from a deeper perspective [135].

All in all metabolomic approaches are still in a continuously growing phase but have the ability to become a valuable tool for herbal medicinal research.

### 6.3. Conclusions

The traditional approach to understand the pharmacology of multi-component agents is based on studying the effect of single components on single biological reactions, enzymes, genes, and so forth and gradually assembling the findings into a whole picture. However, assembling the results obtained from such a reductionist approach to achieve understanding of complex combinatorial pharmacological interactions and pharmacokinetics of multiple components in medicinal plants proves impractical for explaining the observed promising therapeutic effects. Hence neither the pharmacological activity nor the disposition in the body can be attributed to single compound. This is a major obstacle to understanding herbal medicine. Moreover many herbal compounds, as shown before, are known for their poor bioavailability and extensive metabolism in the gastrointestinal tract, where the metabolites rather than the parent compounds are more likely to be absorbed into the circulation system. Therefore the detection and identification of the herbal components absorbed and their metabolites is a crucial step to uncover the pharmacological active substances. For this purpose combinatorial and metabolomic approaches should be taken into consideration when identifying pharmacologically active ingredients and carrying out poly-pharmacokinetic studies on multiple components. The prerequisites are given by an enormous progress of highly sensitive analytical technologies along with the development of multi-variate data analysis methodologies.

Although metabolomics is a relatively new field of science, it has already exhibited considerable potential in herbal medicinal research, or even revolutionized the study of herbal medicines. Nevertheless there are still a lot of challenges to face like further expanding the analytical abilities, improving the understanding of the active ingredient functions and better combining metabolomics with genomics, proteomics, and clinical data. Furthermore standardized operation procedures should be established, including sample processing, reagent selection, data analysis and other details to enable data sharing among different laboratories.

## 7. Other Influence Factors to Be Considered

### 7.1. Physiological Relevance of In Vitro Assays

During preclinical research multiple high-throughput assay approaches are utilized for screening the pharmacological activity of compounds. Often in vitro enzyme activity assays are used to measure the effects of compounds on isolated enzymes. Also immortalized cell-lines are very popular because they are inexpensive, high in throughput, easy to use, reliable, and reproducible. However these model systems, albeit amenable for high-throughput screening (HTS) may not always represent a natural physiological setting and thus may fail to provide exact predictions for the drug in vivo. In contrast, cells contained in human primary whole blood provide a physiologically relevant setting, as well as facilitate assessment of drug protein binding characteristics and the amount of free drug acting on the target. How big the influence of the experimental setting on the results obtained may be is demonstrated on the example of boswellic acids. Hence KBA and AKBA were found to block 5-LO with IC_50_ values of 1.5–50 μM depending on the experimental settings. However in whole blood assays KBA and AKBA failed to inhibit 5-LO product synthesis. Similarly AKBA and KBA failed to suppress PGE_2_ formation in whole blood and in rats despite significant inhibition of mPGES-1 in the cell free assay [29]. The marked loss of activity in whole blood might be related to strong protein binding. A factor that is often not considered when carrying out in vitro assays. Therefore much attention should be given to the experimental settings and their physiological relevance, because inadequate representation of physiological conditions at the preclinical phase can result in inaccurate predictions of compound effects.

### 7.2. Physiological Relevance of In Vitro Absorption Studies in Cell-Line Based Models

Physiological relevance is also a very important issue when carrying out permeability studies. Hence Caco-2 cells obtained from colon carcinoma are considered the gold standard for drug absorption studies. Besides being used alone they are often applied in combination with other cell lines to investigate drug absorption, inflammation, nutrient uptake, and toxicity in the gut as well as in the frame of absorption, distribution, metabolism and excretion studies [138]. However the Caco-2 cells form a non-physiological barrier due to paracellular junctions that are much tighter and less permeable, rendering the Caco-2 model more similar to the colon than to the small intestine [139]. Depending on the laboratory, the clonal type and passage differences transepithelial electrical resistance (TEER) values may vary from 300 to 2400 Ω cm^2^ compared with TEER values of 12 to 120 Ω cm^2^ in human small intestine tissue. This is attributed to the origin of the Caco-2 cells being derived from the large intestine and the smaller average junction pore radius of 3.7 Ǻ compared to 8–13 Ǻ for native human small intestine. In addition Caco-2 cell-based assays suffer from (a) weak expression of important intestinal metabolic enzymes such as cytochrome P450, (b) lack of the crypt-villus axis, which is important for fluid and ion transport, and (c) the absence of mucus producing cells. Another disadvantage of Caco-2 cell-based assays is that the high passages used in many laboratories induce variable expression levels of differentiation markers and transporters [139]. To compensate for these drawbacks a mixed culture of Caco-2 cells and mucus producing methotrexate-treated HT29 colon adenocarcinoma cells mimicking an enterocyte and globlet cell co-culture system has been developed. To further increase the functional complexity of Caco-2 cells triple co-cultures of Caco-2 cells, HT29 cells and Raji B cell line have also been used. However the variability of results between different laboratories could not be reduced by these improvements [138].

A physiologically relevant in vitro intestinal model should ideally closely resemble the human intestinal epithelium structurally and phenotypically comprising the different cell types of the intestinal epithelium. In addition the model should be able to be cultured for long periods [140]. Intestinal explants using porcine intestinal tissue for example as a predictive model for human intestinal absorption suffer from short survival time ex vivo. Small intestinal models generated from normal primary cells isolated from small intestine tissues and intestinal organoids developed from embryonic stem cells or induced pluripotent stem cells recapitulate many of the normal processes of the intestinal mucosa but suffer from disadvantages associated with the physical organization. Hence the villi face inwards which hinders the direct access of test compounds to the apical surface of the enterocytes rendering the conduction of apical-to-basolateral drug permeation studies impossible. While organoids exhibit an inward growth of the luminal surface, 3D organotypic tissue models have an open luminal surface, which makes them ideal for apical application of test compounds mimicking in vivo oral exposure. Full thickness organotypic tissue models comprise intestinal epithelial cells from the ileum, duodenum or jejenum and fibroblasts. They are produced by seeding a mixture of primary small intestinal epithelial cells and fibroblasts onto the microporous membrane of tissue culture inserts under submerged and then at air-liquid-interface for 14 days. In this time, the epithelial cells and fibroblasts self-assemble in the correct orientation and form a distinct and polarized tissue structure with an apical epithelial architecture on top of the fibroblast substrate. Further advantages are the formation of villi, microvilli, brush borders, and tight junctions that mimic the in vivo counterpart. Another attractive feature is that they express efflux transporters and drug-metabolizing enzymes that mimic in vivo intestinal tissues. They can be cultured up to 42 days at an air-liquid-interface and may be utilized for repeat dose application. Reconstruction is possible in 24 single-well culture inserts or in 96-well plate format for high-throughput applications. These tissues have better biological relevance in terms of their potential to simulate drugs absorption, metabolism, toxicity and inflammation. In fact better correlation with human absorption data were observed using organotypic tissues (*r*^2^ = 0.91) compared with Caco-2 cells (*r*^2^ = 0.71). Moreover organotypic tissues allow to study the effect of a substance on isolated intestinal mucosa alone or together with immune cells or in combination with a liver-on-a-chip model rendering them a more holistic model for the investigation of drug absorption and metabolism in human gastrointestinal tract compared with Caco-2 cells. [138].

Another important key element for evaluating drug availability at the site of action is the blood-brain-barrier (BBB) permeability for CNS active herbal components. Unfortunately in vitro experimental designs often fail to take into account binding to the brain tissue as a major factor influencing BBB permeability especially for lipophilic drugs [141]. This might confound the interpretation of data and generate misleading conclusions with regard to the behavior of herbal components in vivo. Traditional methods for assessing the in vitro BBB permeability are based for example on bovine brain capillary endothelial cells (bBCECs) that are seeded on semi-permeable membranes coated with rat tail collagen and placed in a single-well feeding cell culture plate. A simple optimized in vitro model accounting for the effect of binding to brain tissues is based on the presence of glial cells in the receiver compartment to mimic the effect of brain tissue binding in vivo during the permeability experiment. For this purpose the bBCECs are placed on wells seeded with primary cultures of mixed glial cells obtained from the cerebral cortex of new-born rats. The cultures of glial cells are characterized by being composed of 60% astrocytes, 30% oligodendrocytes and 10% microglial cells [108]. The significant improvements of the predictive power observed with the here described simple modification of traditional methods to assess BBB permeability highlights the great influence of tissue binding that is often neglected but should not be underestimated. Therefore tissue binding should be regularly considered for better predicting the availability of herbal components in the CNS as well as integrating in vitro data into physiologically based pharmacokinetic models of CNS drug distribution.

### 7.3. Future Perspectives

In the future further attempts are expected to enhance the physiological relevance of in vitro assays. Even when screening pharmacological effects and absorption of compounds in two-dimensional (2-D) cell-based assays still represent the main pillar of today’s medicinal plant research, it has to be kept in mind that the in vivo complexity of three-dimensional (3-D) tissues cannot be fully recapitulated. It is therefore encouraging to see, that a lot of advances have been already made in the field of physiologically relevant in vitro models including the use of primary cells instead of cell-lines, 3D cell cultures, microtissues and organoids. Recent advances in nanofabrication techniques have led to the development of novel in vitro cell culture models that better mimic the in vivo cellular microenvironement by providing physiologically representative mechanical and structural cues to the cultured cells. Engineered 3-D tissue constructs have been already developed to simulate cardiac, musculoskeletal, skin, lung, kidney, liver, and adipose tissues that are capable of mimicking complex tissue physiology and functionalities [142]. By and by it is expected that tissue-based engineering will be getting cheaper, which will further promote using this fast and ethically more sound technique in medicinal plant research, that enables the use of fewer animals for effective investigations. Furthermore it is envisioned that engineered tissues will be getting even better when combined with advanced computational modeling paving the way for these technologies to become reality in the future. Until then it could be shown in the present review, that even with little modifications to existing experimental designs more physiologically relevant models may be utilized that allow better predictions of the pharmacological activity and absorption behavior of herbal components in vivo.

## 8. Final Conclusions

Because of the complexity of mechanisms involved in most chronic diseases, it is highly questionable that a drug targeting a single gene or enzyme will be sufficiently effective in combating such diseases. Multiple target drugs are gaining thus significant importance. Botanical herbal therapeutics may represent an important source for new drug discoveries as they exert their therapeutic benefits via a multiple-component and multiple-target mode. However this requires much more knowledge about the pharmacological activity of these herbal therapeutics, which is often hampered by the superimposed complexeties of herbal medicines. Even for well-studied herbal plants and substances like *Boswellia serrata*, curcumin, quercetin and resveratrol little is known about their target-site concentrations. This is however very important, because the therapeutic potential of herbal medicine is not only related to its pharmacological activity at the target, but also to its ability to attain an effective dose at the target site. The enormous lack of knowledge regarding biological dispositions is one of the major obstacles to understanding herbal medicines. Therefore novel approaches are needed in medicinal plant research that should focus on combinatorial approaches when evaluating herbal candidates like chemical and metabolomic profiling, ligand fishing and microfluidic techniques. Moreover the physiological relevance of the in vitro models applied should be usually kept in mind in order to avoid inaccurate predictions for the efficacy of herbal components in vivo. In future it is expected that tissue engineering in combination with big data and artificial intelligence will promote the use of combinatorial approaches. This is important as most of the plant components and metabolites likely work in a synergistic fashion or concurrently to give the plant extracts its therapeutic effects. Much effort should be also paid to profile the ingredients of herbal medicines to provide a profound base for pharmacological and bioavailability studies. But it is just as important to adapt the choice of lead substance serving as quality control of herbal extract to the insights gained from physiologically relevant pharmacological and bioavailability studies.

## Figures and Tables

**Table 1:** Overview on the plasma concentrations reported for the individual boswellic acids in pharmacokinetic studies carried out on at least six humans.

Dosage of *Boswellia* Extract	Concentrations of Boswellic Acids in Plasma [µM]						Ref.
	KBA	AKBA	βBA	AβBA	αBA	AαBA	
3 × 4 capsules à 350 mg/day for 1 week (*n* = 14) (in total: KBA 63.6 mg, AKBA 80,4 mg, βBA 2236.8 mg, AβBA 228 mg, αBA 969.6 mg, AαBA 73.2 mg)	0.01–0.52	0–0.03	0.19–26.20	0.26–12.31	0.08–10.59	0.14–5.99	[20]
3 × 282 mg/day—fasted state	0.17 [0.05–0.52]	0.01 [0.002–0.08]	0.4 [0.10–3.9]	ND	ND	ND	[21]
3 × 282 mg/day—fed state (*n* = 12) (in total: KBA 48.12 mg, AKBA 28.71 mg, βBA 143.4 mg, AβBA 82.71 mg, αBA 103.71 mg, AαBA 26.25 mg)	0.48 [0.21–0.9]	0.06 [0.03–0.52]	2.5 [0.91–4.7]	ND in most subjects	0.69 [0.1–2.9]	0.24 [0.09–0.8]	

Values are expressed as range or as mean [range]. ND = not detected.

## Data Availability

Not applicable.
